# Transcriptome analysis reveals the role of GA in fruit development of ‘Cuiguan’ pear (*Pyrus pyrifolia*)

**DOI:** 10.1186/s12870-025-07854-3

**Published:** 2025-12-09

**Authors:** Wenjing Zhai, Xiangzhan Zhang, Yanan Wang, Suke Wang, Yanli Su, Long Wang, Huabai Xue

**Affiliations:** 1 National Key Laboratory for Germplasm Innovation & Utilization of Horticultural Crops, Zhengzhou Fruit Research Institute, Chinese Academy of Agriculture Sciences, Zhengzhou, Henan 453004 China; 2 Zhongyuan Research Center, Chinese Academy of Agriculture Sciences, Xinxiang, Henan 453004 China; 3 Chuxiong Yunfruit Industry Technology Research Institute, Chuxiong, Yunnan 675000 China

**Keywords:** Pear, Gibberellin, Fruit development, Transcriptome

## Abstract

**Background:**

Fruit size is a crucial agronomic trait that significantly influences fruit quality and economic value. Gibberellin serves as an effective phytohormone that promotes fruit expansion and is widely utilized during the early stages of fruit development. However, the mechanisms underlying GA-mediated fruit enlargement in pear, along with the associated genes and regulatory mechanisms, remain poorly understood.

**Results:**

In this study, pear cultivar ‘Cuiguan’ was treated with various combinations of GAs, specifically GA3, GA4 + 7, and GA3 + 4 + 7 at 30 days after flowering. The combination of GA3 + 4 + 7 produced the most pronounced increase in pear fruit size, primarily through promoting elongation. Anatomical analysis revealed that this treatment enhanced both cell proliferation and expansion. RNA-seq was conducted and identified differentially expressed genes associated with GA3 + 4 + 7-mediated fruit enlargement. Functional annotation and enrichment analyses highlighted the involvement of genes related to plant hormone signaling, cell growth, cell division, cell cycle, and transcription factors. Nine DEGs associated with fruit size were validated by RT-qPCR analysis, confirming the RNA-seq results.

**Conclusions:**

This study elucidates the molecular mechanisms by which gibberellins regulate pear fruit development and identify key genes involved in this process, providing a foundation for future research on GA3 + 4 + 7-mediated fruit size regulation.

**Supplementary Information:**

The online version contains supplementary material available at 10.1186/s12870-025-07854-3.

## Background

Fruit size is a crucial determinant of fruit quality and significantly influences the economic value of most horticultural crops. Larger-sized fruits have long been a primary target of human selection throughout the processes of plant domestication and improvement. This preference is largely attributed to the fact that larger fruits typically offer higher quality, increased productivity, and greater consumer acceptance, ultimately leading to greater economic benefits.

Fruit size is a complex trait influenced by an intricate interplay of genetic, environmental, and physiological factors. This is especially suitable for pears, which develop primarily from the receptacle of the flower and the base of the floral tube. Fruit development is affected by events occurring both before and after flower opening. Before flowering occurs, the number of cells that will contribute to fruit formation. Following flower opening, two distinct phases determine the final fruit size: cell division and cell enlargement.

Genetic factors play a crucial role in determining fruit size. Research on fruit size has been conducted across various plant species, including cherries, tomatoes and *Physalis*. In sweet cherries, *PaCYP78A9*, a member of the cytochrome P450 subfamily, influences fruit size by mediating pericarp cells division and expansion [1]. In tomatoes, it has been discovered that regulating the expression of key factors within plant cyclin-dependent kinases (CDKs) can alter the balance between cell division and cell expansion, thereby affecting overall fruit size [2]. In *Physalis*, the *POS1* gene is associated with variations in fruit size; *POS1* engages in several regulatory pathways to modulate cell expansion, thus regulating fruit size [3]. Furthermore, multiple molecular mechanisms have been identified as critical factors that coordinately regulate fruit development. These include transcription factors, quantitative trait locus (QTL), the CLV-WUS signaling pathway, the ubiquitin–proteasome pathway, microRNA pathways, among others [4–6].

Numerous additional factors also contribute to fruit development, including environmental conditions, internal plant dynamics and horticultural practices. Furthermore, the application of phytohormones and other treatments can directly modulate fruit size. Various phytohormones, including cytokinin, auxin, gibberellin, and abscisic acid, have been reported to regulate fruit size across different fruit crop species, such as apples, tomatoes and peaches. Among these hormones, cytokinin [7], auxin, and gibberellin [8] have been identified as promoters of fruit enlargement. In contrast, abscisic acid inhibits fruit growth [9], while ethylene leads to a reduction in fruit weight [10].

Previous studies have demonstrated the significant impact of gibberellins on fruit development. For example, the application of GA3 in tomatoes not only induces parthenocarpy but also enhances fruit quality, resulting in increased sugar content and reduced acidity [11]. In sweet cherries, GA4 serves as an effective artificial dormancy-breaking agent, reducing the time required for the sprouting of flower buds and improving their sprouting rate [12]. The biosynthetic pathways of gibberellin metabolism and signal transduction are well established, involving a variety of enzymes [13]. The key enzymes responsible for the initial stages of GA synthesis include ent-copalyl diphosphate synthase (CPS), ent-kaurene synthase (KS), ent-kaurene oxidase (KO), and ent-kaureneoic acid oxidases (KAO). These enzymes catalyze the formation of GA12-aldehyde, which then progresses through two distinct pathways-hydroxylation and non-hydroxylation-to yield GA53 and GA12, respectively. Subsequently, both GA12 and GA53 are further oxidized by GA20ox and GA3ox within the cytoplasmic matrix to produce various active gibberellins. Additionally, GA2ox is responsible for the catabolic breakdown of active GAs. The gibberellin signaling pathway consists of two main components: cellular perception of gibberellin signals and subsequent cellular responses elicited by these signals. The gibberellin receptor known as GA insensitive dwarf 1 (GID1) is pivotal for detecting gibberellin signals and facilitating their binding. The DELLA protein functions as a negative regulator in controlling the expression levels of genes responsive to gibberellin. The SCF ubiquitin ligase E3 complex contributes to the degrading of DELLA protein, thereby alleviating their inhibitory effects. As active GAs concentrations increase within plants, the expression of gibberellin synthesis genes such as GA20ox and GA3ox is inhibited, while the expression of GA2ox, involved in gibberellin metabolism, is promoted [14]. When gibberellins are present at elevated concentrations, the gibberellin receptor GA insensitive dwarf 1 (GID1) binds to GA, and the DELLA protein modulates the transcription of downstream target genes through ubiquitination, thereby promoting plant growth and development [15, 16]. Consequently, gibberellin signaling pathways can significantly influence fruit development in horticultural plants.

Despite the progress made in understanding fruit size regulation across various plant species, there remains a notable gap in research exploring the effects and underlying molecular mechanisms of different gibberellin species on fruit development. In this research, various GAs were applied to the pear cultivar ‘Cuiguan’ to investigate their effect on fruit size. Transcriptome analysis was conducted on GA3 + 4 + 7-treated pears and the corresponding control group to identify pathways and key genes associated with fruit size. Alongside annotated genes related to fruit size and differential expressed genes, several candidate genes linked to fruit size were selected for RT-qPCR analysis. This research provides valuable insights for future studies focusing on GA-mediated regulation of fruit size, shedding light on the mechanisms governing pear fruit size regulation.

## Methods

### Plant materials and experimental treatments

Pear cultivar ‘Cuiguan’ (*Pyrus pyrifolia*) was used as an experimental material. Six-year-old pear trees were planted in the orchard of Zhengzhou Fruit Research Institute (34°72′N 113°71′E), Chinese Academy of Agricultural Sciences, and managed under standard agricultural practices. Five pear trees with similar growth conditions were selected for both treatments and corresponding control groups.

For the gibberellic acid (GA) treatment, various GAs compounds were dissolved in alcohol and mixed with lanolin as a carrier medium. Treatments were administered 30 days after full blooming (DAFB), the fruit stalks were treated with 2.7% (g/g) GA3, 2.7% (g/g) GA4 + 7 and 2.7% (g/g) GA3 + 4 + 7 respectively. Alcohol mixed with lanolin served as the control group.

### Phenotypic and physiological analysis

Pear fruits were sampled at 0, 10, 20, 30, 40, 50, 60, 70 and 80 days after treatment (DAT). Phenotypic measurement and physiological analyses were performed on the following traits, including vertical diameter (cm), horizontal diameter (cm), fruit shape index (calculated as the ratio of longitudinal diameter to horizontal diameter), and single fruit weight (g). At least five biological replicates should be performed for each treatment and at each time point.

### Paraffin sectioning

For paraffin sectioning, vertical sections of flesh tissue (approximately 1 cm from the fruit peel) were collected at 0, 10, 20, 30, 40, 50, 60, 70 and 80 DAT. Three biological replicates should be performed for each treatment and at each time point. Pear samples were immediately fixed in a fixative composed of formaldehyde-ethanol-glacial acetic acid (70% ethanol: formalin: glacial acetic acid = 18:1:1). The flesh tissues were dehydrated using a gradient series of alcohol before being embedded in paraffin. Paraffin blocks were sliced using a Donatello microtome (DIAPATH, Tuscany region, Florence, Italy). Subsequently, the paraffin sections were stained with fast green (1% w/w) and safranin (1% w/w). Images of flesh cells were captured and observed using a VHX-6000 digital microscope (KEYENCE, Osaka, Japan). Nine fields of view were selected for each developmental stage, and cell counts were performed using Image J software (National Institute of Health, Bethesda, USA).

### RNA Extraction, cDNA library Preparation, and RNA sequencing

Pulp tissues of the pears were collected at 0, 12, and 24 h after treatment, and then frozen in liquid nitrogen and stored at −80℃, followed by RNA-seq analysis. Three biological replicates should be performed for each treatment and at each time point. Total RNA was extracted from flesh samples using an RNA Extraction Kit (ZOMANBIO, Beijing, China) according to the manufacturer’s instructions. RNA integrity was assessed by agarose gel electrophoresis and further verified using an Agilent 2100 Bioanalyzer (Agilent Technologies, Palo Alto, CA, USA). cDNA libraries were sequenced on the Illumina sequencing platform (Illumina, San Diego, CA, USA) by Genedenovo Biotechnology Co., Ltd (Guangzhou, China). Raw reads were filtered using fastp [17] to obtain clean reads, which were subsequently mapped to the *Pyrus pyrifolia* Cuiguan Genome v1.0 (https://www.rosaceae.org/Analysis/11815273, accessed on 27 September 2023)using HISAT2.2.4 [18].

### Differentially expressed gene (DEG) analysis and functional annotation

Based on the alignment from HISAT2, the transcripts were reconstructed using Stringtie [19]. Gene expression levels were quantified using RSEM [20] and presented as fragment per kilobase of transcript per million mapped reads (FPKM). Principal component analysis (PCA) and Pearson correlation coefficient calculations were performed using the R package, along with heatmap generation in R software. Differential gene expression analysis was conducted using DESeq2 [21], and significant DEGs were identified based on the false discovery rate (FDR) value < 0.05 and |log2(fold change)|>1. Volcano plots illustrating DEGs among various groups were generated using the ggplot2 package in the R software environment. Gene ontology (GO) enrichment analysis of these DEGs was performed referencing the GO database (http://www.geneontology.org/, accessed on 18 October 2023). Pathway enrichment analysis was executed employing the Kyoto Encyclopedia of Genes and Genomes (KEGG) database (https://www.kegg.jp/, accessed on 18 October 2023) with p-value < 0.05 considered statistically significant.

### RNA isolation and RT-qPCR analysis

Flesh samples were frozen in liquid nitrogen and ground into powder. Total RNA was exacted using an RNA Extraction Kit (ZOMANBIO, Beijing, China) following the manufacturer’s protocol. cDNA synthesis was performed using the TransGen One-Step gDNA Removal and cDNA Synthesis SuperMix (TransGen Biotech, Beijing, China). RT-qPCR analysis was conducted using the TransStart Top Green qPCR SuperMix (TransGen Biotech, Beijing, China) on the Roche LightCycler 480 system (Roche, Basel, Switzerland). All RT-qPCR were conducted in triplicate, with the pear *PcTubulin* gene as an internal control. Relative expression levels were calculated using the 2^−∆∆Ct^ method [22]. Specific primers used for RT-qPCR analysis are detailed in Table S1.

### WGCNA and gene Co-Expression network construction

Weighted gene co-expression network analysis was performed utilizing DEGs identified based on an FDR threshold of < 0.05 and |log2(fold change)|>1. The co-expression network was constructed with hub genes associated with fruit size development within the indicated module. The gene co-expression network was visualized using Cytoscape (v3.5.1, Paul Shannon, Seattle, WA, USA).

## Results

### Impact of gibberellins on the growth and development of Pear fruits

**Fig. 1:**
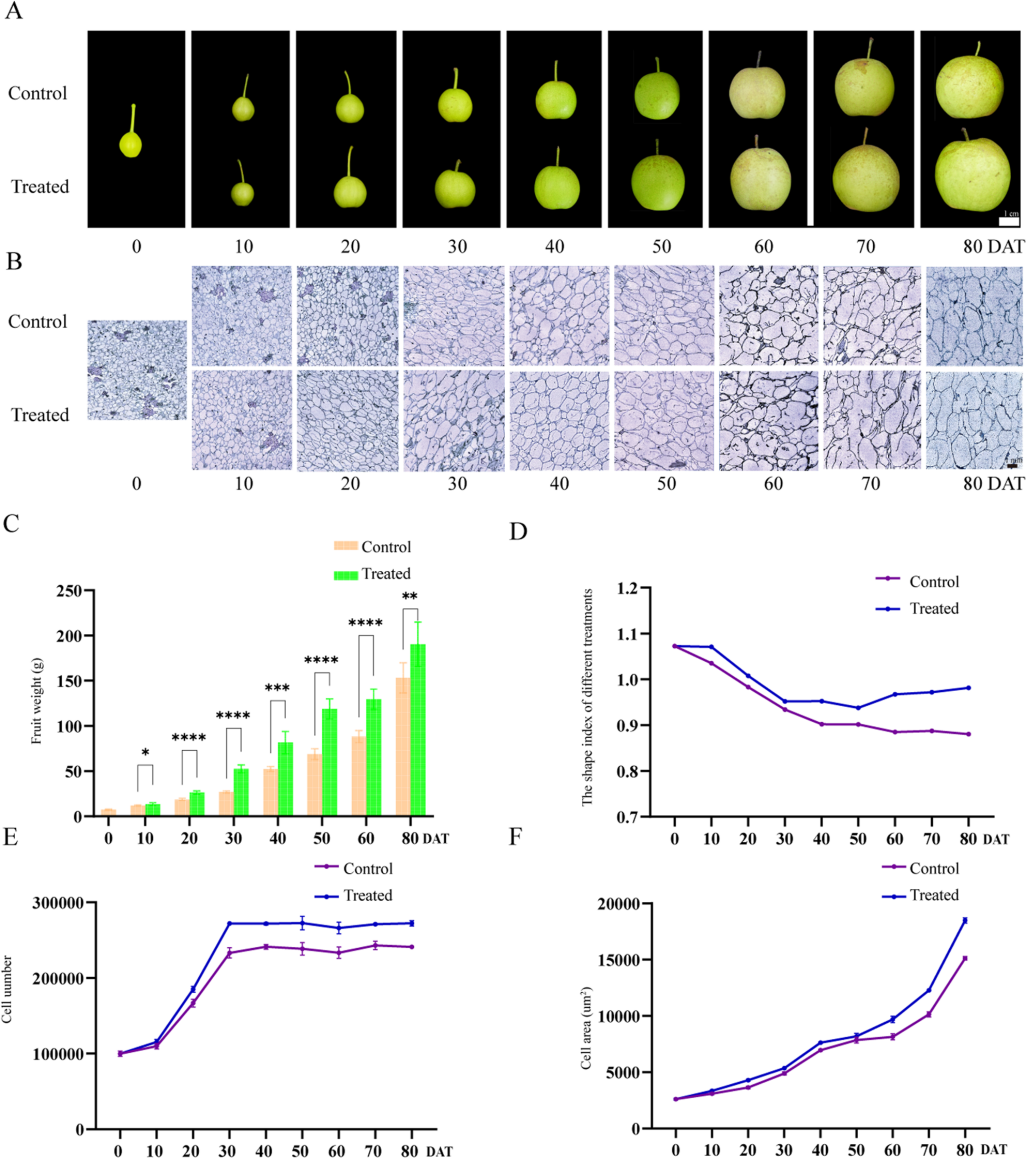
Phenotypic characteristics of pear fruit treated with exogenous GA3 + 4 + 7 from 0 to 80 DAT. **A** Morphological features illustrate the growth and development status of pear fruit treated with exogenous GA3 + 4 + 7 from 0 to 80 DAT. Bar = 1 cm. **B** Paraffin section of the flesh of pear fruit subjected to GA3 + 4 + 7 treatment and its corresponding control. Bar = 1 mm. **C** Single fruit weight (Fw) measurements of pear fruits treated with exogenous GA3 + 4 + 7 from 0 to 80 DAT. **D** Fruit shape index of pear fruits treated with exogenous GA3 + 4 + 7 from 0 to 80 DAT. **E** Cell number of pear fruits treated with exogenous GA3 + 4 + 7 from 0 to 80 DAT. **F** Cell area of pear fruits treated with exogenous GA3 + 4 + 7 from 0 to 80 DAT

To investigate the effects of different gibberellins (GAs) compounds on fruit development, GA3, GA4 + 7, and GA3 + 4 + 7 were applied to the pear cultivar ‘Cuiguan’ 30 DAFB. The transverse and longitudinal diameters of pear fruits were measured at multiple time points, including 0, 10, 20, 30, 40, 50, 60, 70, and 80 DAT. Results indicated that GA3 + 4 + 7 treatment significantly enhanced both the transverse and longitudinal diameters of ‘Cuiguan’ fruits compared to controls and other GA-treated groups, with the most pronounced effect observed throughout the developmental period (Fig. S1). Additionally, GA3 + 4 + 7 treatment led to a substantial increase in single fruit weight at all evaluated time points from 10 to 80 DAT, highlighting its positive influence on fruit size enlargement (Fig. 1A, C). Analysis of the fruit shape index revealed a continuous decline throughout the entire fruit developmental stage with significantly higher values observed in the GA3 + 4 + 7-treated group compared to the control group (Fig. 1D; Table S2). This suggests that GA3 + 4 + 7 promotes longitudinal elongation of the fruit, thereby affecting overall fruit morphology.

Additionally, paraffin sectioning of flesh cells was performed at various developmental phases. Results showed that GA3 + 4 + 7 treatment enhanced cell numbers and increased cell size compared to controls. Specifically, cell numbers increased consistently during the first 30 DAT, reaching a plateau until maturity. Cell size remained relatively stable during the initial 30 DAT following GA3 + 4 + 7 treatment, before expanding continuously until the ripening stage. Together, these results demonstrate that the exogenous application of GA3 + 4 + 7 effectively enhances pear fruit size, primarily by promoting longitudinal elongation. Both cell number and cell size contribute to the final fruit dimensions, with GA3 + 4 + 7 treatment significantly influencing these parameters (Fig. 1B, E, and F).

### Transcriptome analysis of Pear following GA3 + 4 + 7 treatment

**Fig. 2:**
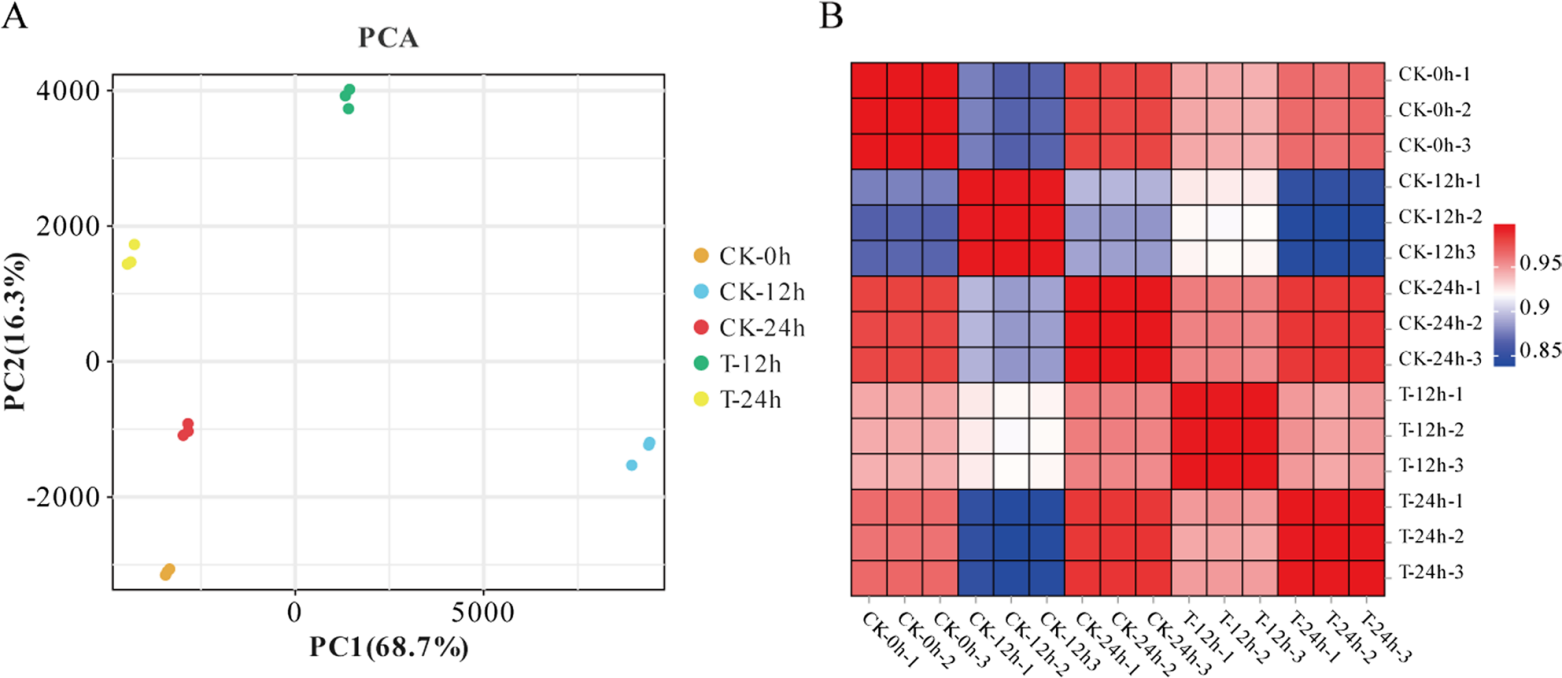
Relationships between samples from the GA3 + 4 + 7-treated group and their corresponding controls at various time points. **A** Principal Component Analysis (PCA) of samples from the GA3 + 4 + 7-treated group compared to their respective controls across different time points. **B** Pearson correlation coefficient analysis for samples from the GA3 + 4 + 7-treated group versus their corresponding controls at distinct time intervals. The intensity of red coloration indicates a stronger similarity among samples. CK refers to the control group, while T represents the GA3 + 4 + 7-treated group.

To elucidate the transcriptional mechanisms underlying GA3 + 4 + 7-mediated fruit development, RNA-seq was conducted on ‘Cuiguan’ fruits treated with GA3 + 4 + 7 at 30 DAFB. Three time points (0, 12, and 24 h) were selected for analysis, with three biological replicates for each time point. The sequencing data exhibited high quality, with Q20 values ranging from 96.45% to 97.96%, and Q30 values from 92.53% to 96.23%. The mean GC content was 46.65% (Table S3), and the average alignment rate to the *Pyrus pyrifolia* Cuiguan Genome v1.0 was 90.44% (Table S4). Principal component analysis (PCA) indicated that biological replicates clustered closely together across different time points, indicating high consistency among replicates (Fig. 2A). The Pearson correlation analysis indicates a positive correlation among the samples, reflecting the reliability of the experiment (Fig. 2B).

**Fig. 3:**
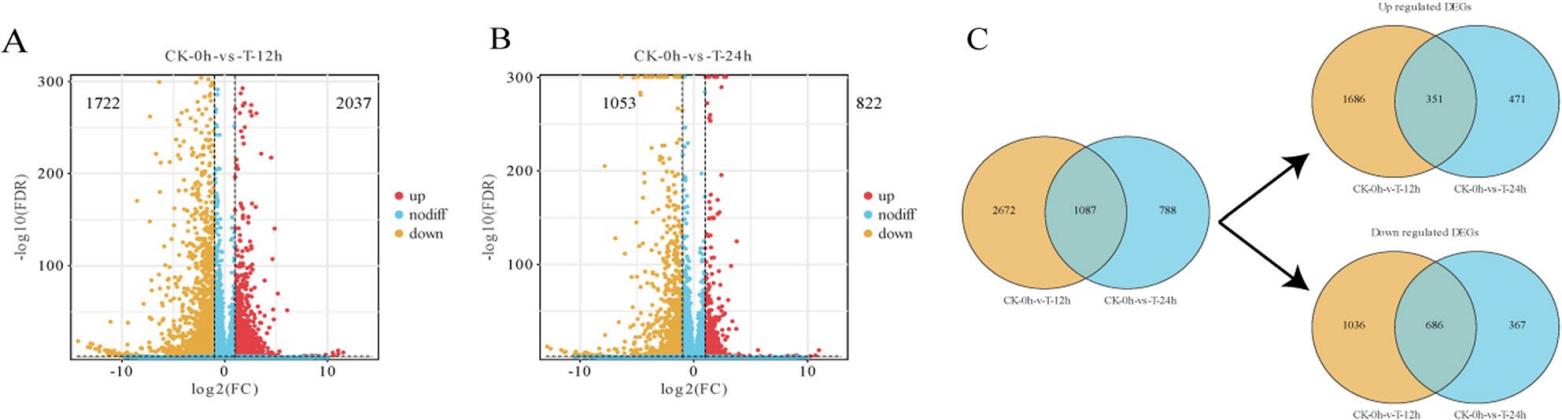
Differentially expressed genes (DEGs) in response to GA3 + 4 + 7 treatment. **A **DEGs identified between 0 h and 12 h post treatment. **B** DEGs identified between 0 h and 24 h post treatment. **C** Venn diagram illustrates the overlap of DEGs between the 12 h and 24 h time points. Red dots indicate up-regulated genes, orange dots denote down-regulated genes, and blue dots represent genes with no significant difference in expression levels. The counts of up-regulated and down-regulated DEGs are displayed in the corresponding Figs. CK refers to the control group, while T represents the GA3 + 4 + 7-treated group

To identify candidate genes responsive to GA3 + 4 + 7 treatment, DEGs were defined based on an FDR threshold of less than 0.05 and |log2(FC)| greater than 1. A total of 2,037 up-regulated DEGs and 1,722 down-regulated DEGs were identified between the 0 h and 12 h time points following GA3 + 4 + 7 treatment (Fig. 3A). Additionally, 822 up-regulated DEGs and 1,053 down-regulated DEGs were identified between the 0-hour and 24 h groups (Fig. 3B). Overall, 1,087 DEGs were shared between the two groups, including 351 consistently up-regulated DEGs and 686 consistently down-regulated DEGs (Fig. 3C).

Compared to the control group, GA3 + 4 + 7 treatment resulted in significant changes in gene expression levels. After 12 h of GA3 + 4 + 7 treatment, 1,187 up-regulated DEGs and 1,632 down-regulated DEGs were identified when compared with the control at 12 h post GA3 + 4 + 7 treatment (Fig. S2). Following 24 h of GA3 + 4 + 7 treatment, 417 up-regulated DEGs and 214 down-regulated DEGs were detected when compared with the control at 24 h post GA3 + 4 + 7 treatment (Fig. S2). These results indicate that GA treatment induces profound alterations in the transcriptional profiles of pear fruits, with many genes showing significant changes in expression levels.

### Functional analysis of the differentially expressed genes

**Fig. 4:**
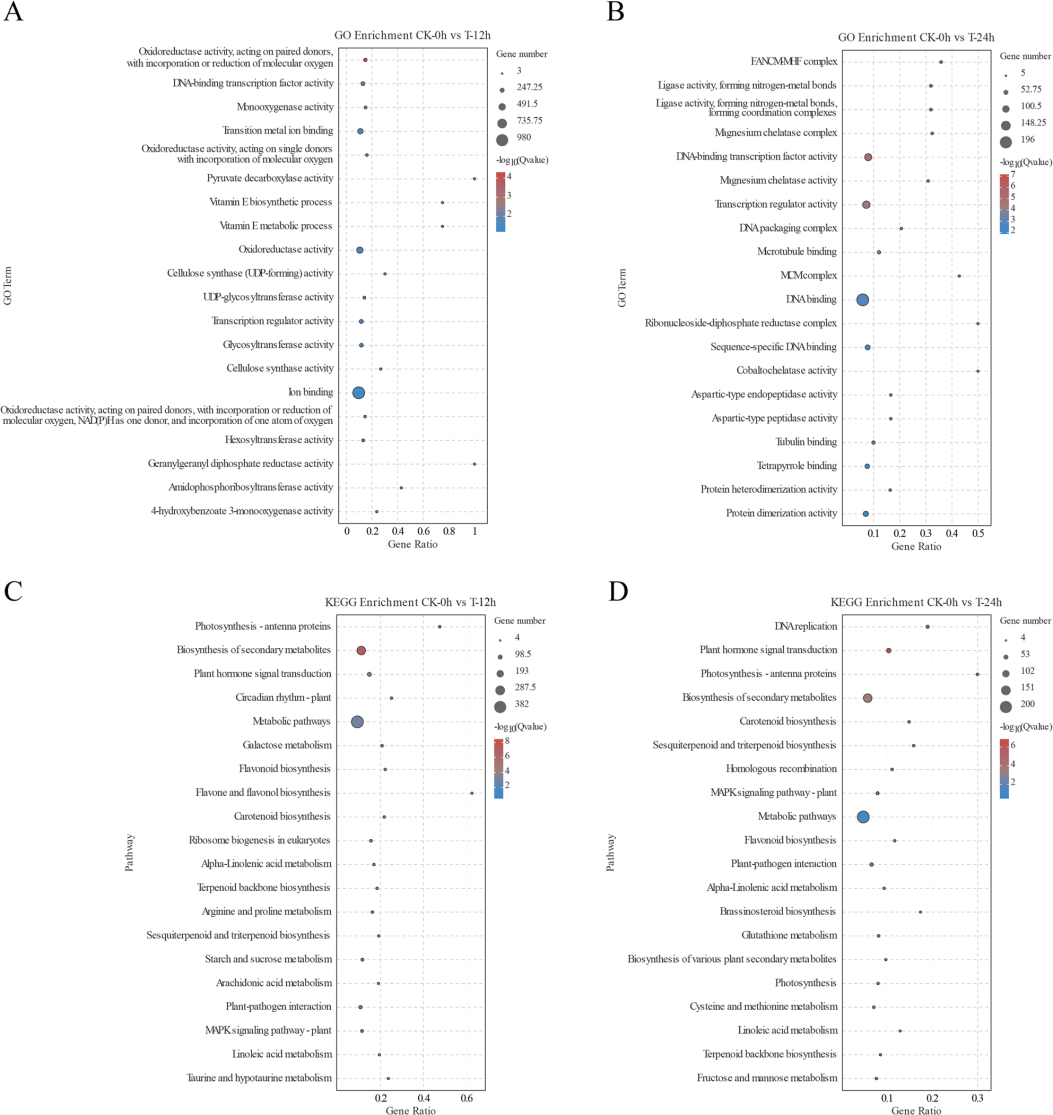
GO and KEGG enrichment analysis of DEGs between GA3 + 4 + 7-treated and control groups. **A **GO enrichment analysis comparing the control group (CK) at 0 h with the GA3 + 4 + 7-treated group (T) at 12 h. **B** GO enrichment analysis comparing CK at 0 h with the GA3 + 4 + 7-treated group (T) at 24 h. **C** KEGG enrichment analysis comparing CK at 0 h with the GA3 + 4 + 7-treated group (T) at 12 h. **D** KEGG enrichment analysis comparing CK at 0 h with the GA3 + 4 + 7-treated group (T) at 24 h. Note: CK represents the control group, while T denotes GA3 + 4 + 7-treated group

To elucidate the potential pathways associated with the DEGs identified in various comparisons, a Gene Ontology (GO) enrichment analysis was performed using the corresponding DEGs. The GO enrichment analysis of GA3 + 4 + 7 treatment at 12 h and 24 h compared to 0 h revealed that the most significantly enriched terms were associated with catalytic activity and binding categories. Notable enriched categories included ‘oxidoreductase activity’, ‘DNA-binding transcription factor activity’, ‘monooxygenase activity’, ‘transition metal ion binding’, ‘transcription regulator activity’ and ‘ligase activity’ (Fig. 4A, B). Furthermore, categories related to cell wall synthesis, such as ‘cellulose synthase (UDP-forming) activity’ and ‘cellulose synthase activity’, as well as those involved in DNA replication including ‘DNA-binding’ ‘DNA packaging complex’, and ‘hexosyltransferase activity’, which is implicated in plant hormone synthesis, were highly enriched. These findings may be correlated with fruit size development. Comparative GO enrichment analyses were also performed between the control and GA3 + 4 + 7-treated groups at 12 h (CK-12 h vs. T-12 h) and 24 h (CK-24 h vs. T-24 h). The results revealed significant enrichment of GO terms in both groups, including ‘integral component of membrane’, ‘cell cycle DNA replication initiation’, ‘nuclear cell cycle DNA replication initiation’, ‘protein kinase activity’ and ‘hexosyltransferase activity’ (Fig. S3).

To further investigate the potential functional pathways involved in GA-mediated fruit development, KEGG enrichment analysis was performed utilizing DEGs from various comparisons. The results indicate that, when comparing pathways at 0 h with those at 12 h and 24 h post GA3 + 4 + 7 treatment, significant enrichments were observed in several pathways, including ‘Biosynthesis of secondary metabolites’, ‘Plant hormone signal transduction’, ‘Flavonoid biosynthesis’, ‘Carotenoid biosynthesis’, ‘Sesquiterpenoid and triterpenoid biosynthesis’, ‘MAPK signaling pathway-plant’ and ‘DNA replication’ (Fig. 4C, D). These findings suggest that GA-mediated fruit development likely involves these metabolic pathways, with GA treatment potentially influencing DNA replication and plant hormone signal transduction.

Additionally, KEGG enrichment analysis was performed on DEGs from other comparison groups, including CK-12 h vs. T-12 h and CK-24 h vs. T-24 h. The results demonstrated that DEGs within these groups exhibited similar and overlapping metabolic pathways, with significant enrichments identified in ‘Biosynthesis of secondary metabolites’, ‘Plant hormone signal transduction’, ‘DNA replication’, ‘Carotenoid biosynthesis’, and ‘Sesquiterpenoid and triterpenoid biosynthesis’ across different groups. (Fig. S4).

### DEGs involved in fruit size development

**Fig. 5:**
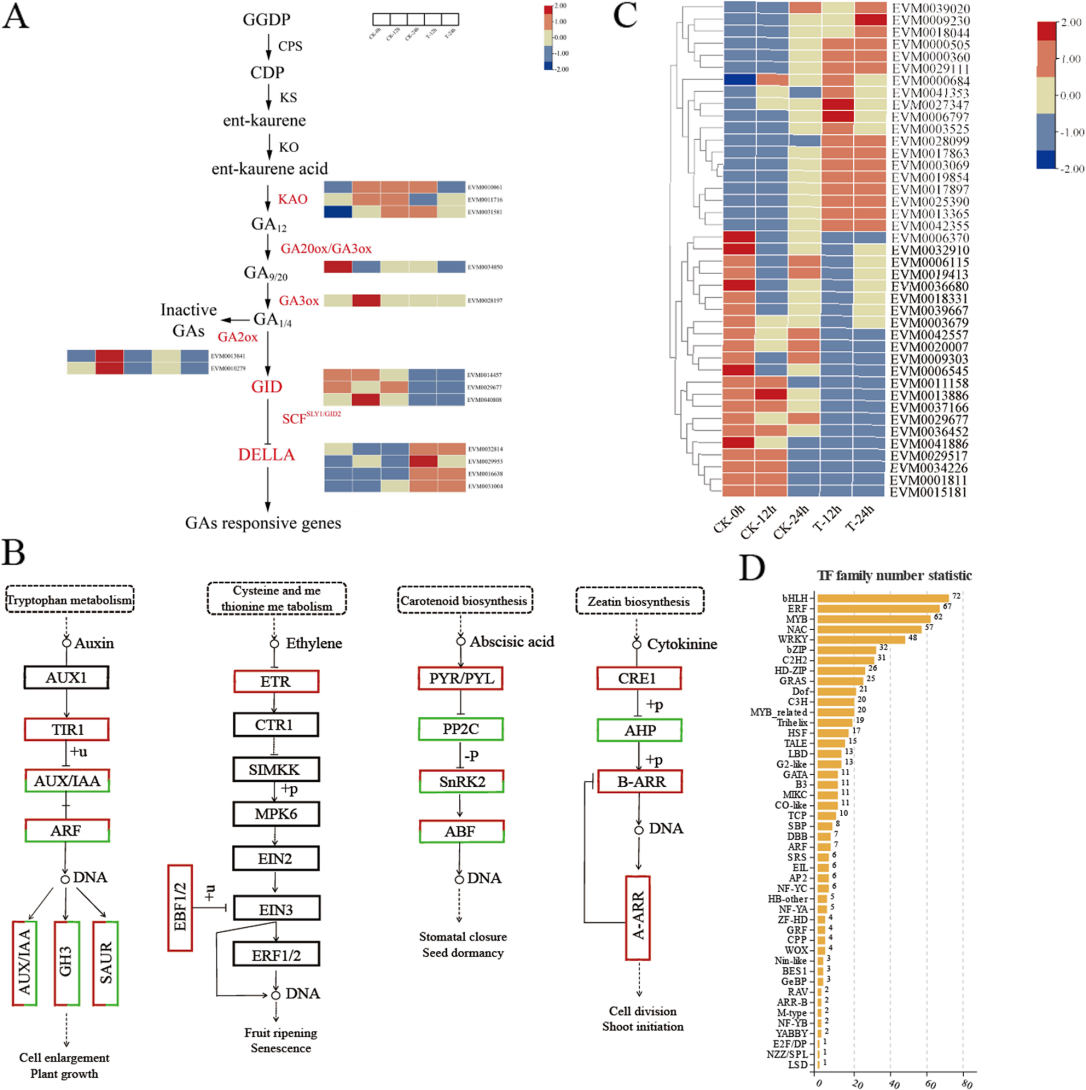
Characterization of genes and regulators related to fruit size. **A** Heatmap of gene expression profiles in GA biosynthesis and signaling pathways following GA3 + 4 + 7 treatment. **B** Simplified sketch of hormone signaling pathways, with red shadow indicating up-regulated enzymes and green shadows indicating down-regulated ones. **C** Heatmap of fruit size-related genes under GA3 + 4 + 7 treatment. **D** The number of differentially expressed TF genes between the treated samples and their corresponding control. The color scale indicates gene expression levels, with red representing high expression and blue represents low expression levels

To elucidate the transcriptional changes of genes associated with GA synthesis and signal transduction, a total of 66 genes were identified based on prior reports and the gene annotations (Table S5). An FDR threshold of less than 0.05 and |log2 (FC)| greater than 1 were applied for selection criteria, resulting in the identification of 14 DEGs between treatment and control groups. (Fig. 5A).

Among the genes associated with GA biosynthesis, *PyKAO1-like* (*EVM0011716*) and *PyGA20ox1-like* (*EVM0034050*), exhibited significantly reduced expression levels following GA treatment. In contrast, *PyGA2ox1-like* (*EVM0013841* and *EVM0010279*), a key enzyme involved in the degradation of active GA, was found to be up-regulated in the GA-treated group compared to the control group. Additionally, *PyGA3ox1-like* (*EVM0028197*), an essential gene within the biosynthetic pathway of active GA, demonstrated down-regulation in the T-12 h group relative to the CK-12 h group.

The GA receptor GID (*PyGID1B*), F-box protein (*PyGID2*), and DELLA proteins (*PyGAI* and *PyGAI1*) are integral components of the GA signaling pathway. Comparisons between both CK-0 h and T-12 h groups as well as between CK-0 h and T-24 h groups revealed that both the *PyGID1B-like* (*EVM4000808* and *EVM0014457*) and *PyGID2-like* (*EVM0029677*) were found to be down-regulated. Similarly, DELLA proteins such as PyGAI-like (*EVM0031004*)、PyGAIPB-like(*EVM0016638*) PyGAI1-like (*EVM0029953* and *EVM0032814*) displayed upregulation in these comparisons. These findings indicate that exogenous GA treatment significantly induces the expression of key genes associated with GA signaling, thereby underscoring the critical role of GA in modulating gene expression.

To identify potential genes associated with phytohormone signal transduction that interact with GA application-mediated fruit development, the transcript of genes associated with various phytohormone signaling pathways were analyzed (Fig. 5B). It was revealed that in ‘Cuiguan’, genes encoding receptor proteins transport inhibitor response 1 (TIR1), AUXIN/Indole-3-acetic acid (AUX/IAA), auxin response factor (ARF), gretchen Hagen 3 (GH3), and small auxin-up RNA (SAUR) exhibited differential expression following the application of GA3 + 4 + 7(Fig. 5B). Thus, the application of exogenous gibberellins (GA) may trigger the expression of key genes in the auxin signaling pathway, such as the auxin response factor IAA12 (EVM0018389) and GH3.1 (EVM0039583) (Fig. S5).

Additionally, within the ethylene signaling pathway, the ethylene receptor (ETR) and EIN3-binding F-box protein 1/2 (EBF1/2) have been identified, with EBF1/2 functioning as a negative regulatory factor. This suggests that the exogenous application of gibberellins (GA3 + 4 + 7) may potentially attenuate the ethylene signaling pathway.

Furthermore, in the cytokinin signaling pathway, differential expressions were noted for histidine-containing phosphotransfer protein (AHP) and arabidopsis response regulator (A-ARR). In contrast, within the abscisic acid signaling pathway, DEGs included abscisic acid receptor pyrabactin resistance 1-like (PYR/PYL), protein phosphatase 2 C (PP2C), sucrose non-fermenting related protein kinase (SnRK), and ABA responsive element binding factor (ABF).

These results suggest that exogenous treatment with GA significantly induces gene expression related to auxin, cytokinin and abscisic acid pathways-potentially facilitating crosstalk with GA-mediated fruit development. Considering that GA treatment significantly increased pear fruit size and coupled with the fact that fruit size is determined by both cell size and number, the DEGs associated with cell division and expansion were identified through the transcriptome analysis. These include the cytochrome P450 genes, F-box proteins, kinesins, genes involved in cell expansion (EXP), cell wall-modifying enzyme xyloglucan endotransglucosylase/hydrolase (XTH), cyclin-dependent kinases (CDKs), cyclin genes, tonoplast intrinsic proteins(TIPs), and various membrane proteins (Fig. 5C; Table S6).

Among these DEGs, after GA3 + 4 + 7 application for 12 h and 24 h, we observed up-regulation of cytochrome P450-like genes (*EVM0000360* and *EVM0017897*), F-box protein (*EVM0000505* and *EVM0017863*), kinesin-like protein (*EVM0003069*, *EVM0009230*, *EVM0013365*,* EVM0018044* and *EVM0028099*), cyclin-like genes (*EVM0019854*, *EVM0025390*, and *EVM0029111*), XTH protein (*EVM0041353*), as well as TIP-like (*EVM0042355*).

Conversely, several other cytochrome P450-like genes (EVM0001811, EVM0003679, EVM0009303, EVM0015181, EVM0020007, EVM0034226 and EVM0042557), F-box protein (EVM0011158, EVM0013886, EVM0029517, EVM0029677, EVM0037166 and EVM0041886), expansion protein (EVM0006115, EVM0018331, EVM0019413, EVM0032910 and EVM0039667), membrane protein (EVM0036680), and xyloglucan endotransglucosylase/hydrolase protein-like (EVM0036452) were found to be down-regulated. The results indicate that these specific genes may play a crucial role in regulating pear fruit enlargement by coordinating processes of cell division and cell expansion.

Transcription factors (TFs) are essential for the spatiotemporal regulation of plant growth and development. In our study, we identified a total of 702 TFs belonging to 46 distinct families (Fig. 5D). The ten most abundant TF families, in terms of quantity, include bHLH, ERF, MYB, NAC, WRKY, bZIP, C2H2, HD-ZIP, GRAS, and Dof. Notably, these TFs exhibit differential expression following treatment with exogenous GA3 + 4 + 7, suggesting their significant role in transcriptional regulation during fruit development.

### Co-Expression networks of genes associated with fruit size

**Fig. 6:**
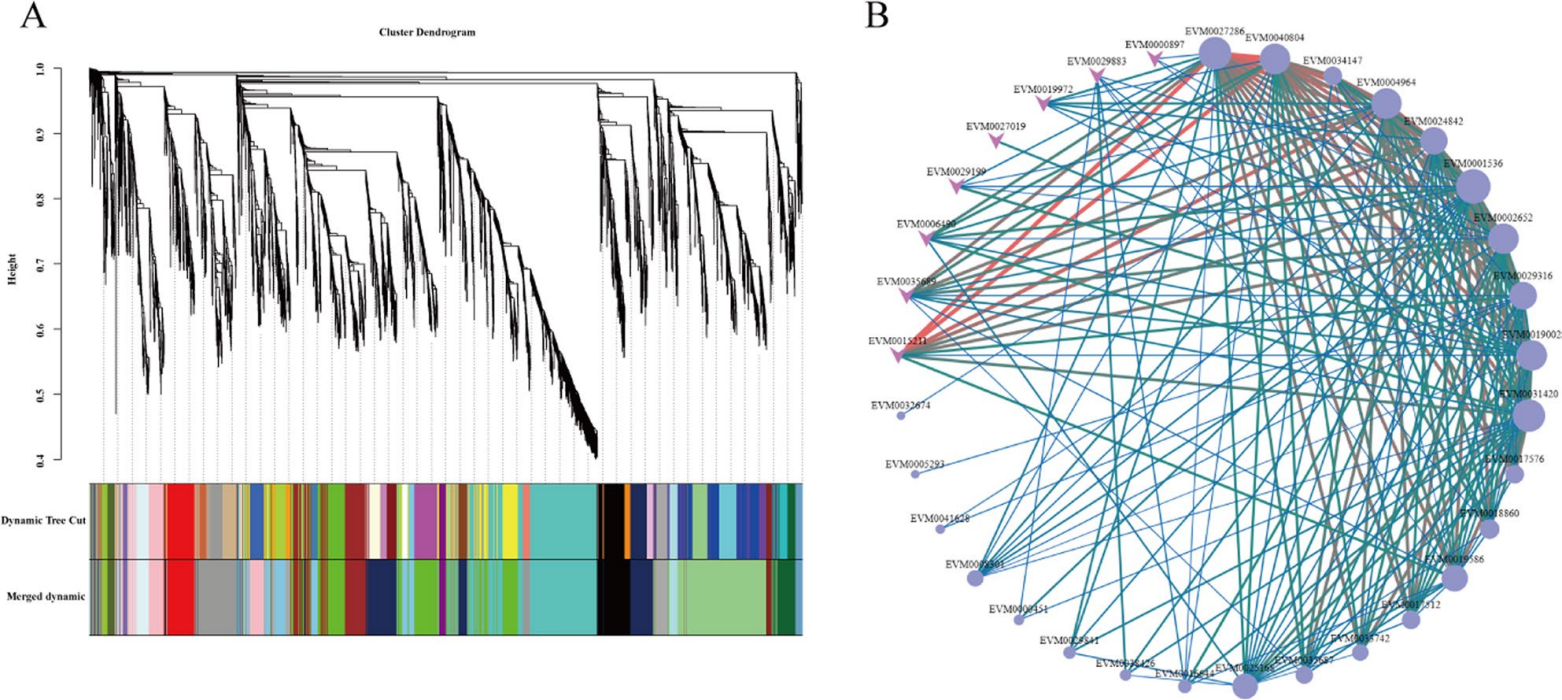
Identification of core genes related to fruit size in the co-expression network. **A** Hierarchical clustering dendrogram of 21 co-expressed gene modules. Each leaf on the tree represents an individual gene. **B** The co-expression network features 34 MM.black module genes. Circles denote structural genes, arrows indicate transcription factors, node size reflects gene connectivity, and line thickness and color represent weight values

To investigate the relationships among genes associated with fruit size mentioned above, a weighted gene co-expression network analysis (WGCNA) was conducted with all DEGs. The analysis revealed 21 distinct co-expression modules (Fig. 6A). KEGG enrichment analysis indicated that the black module was significantly enriched in pathways related to DNA replication and plant hormone signal transduction. Within the black module, a total of 22 genes associated with DNA replication, nine genes involved in plant hormone signaling pathways, and nine bHLH transcription factors that exhibited the highest abundance. Based on the weight values and the connectivity of candidate genes within this, 34 key genes were ultimately selected for constructing the co-expression network (Fig. 6B). Collectively, our comprehensive analysis of the WGCNA results suggests that the MM.black module serves as a primary gene co-expression network containing these 34 pivotal candidate genes most closely related to fruit size. This implies that these genes are likely implicated in GA-regulated fruit development and exhibit interconnectedness with one another.

### RT-qPCR analysis of genes involved in Gibberellin biosynthesis and signaling pathways

**Fig. 7:**
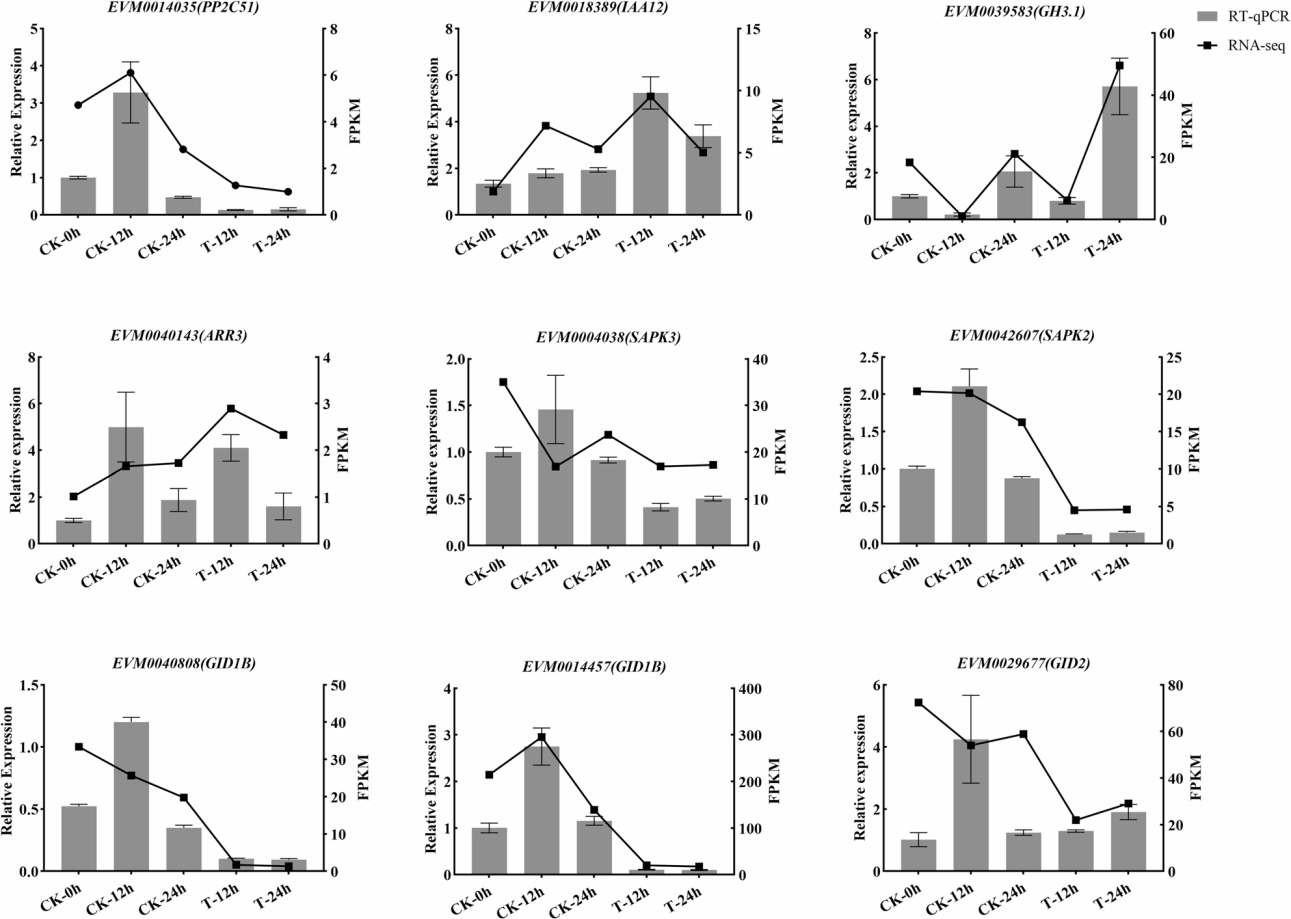
RT-qPCR analysis of plant hormone-related genes under GA3 + 4 + 7 treatment

To validate the transcriptomic alterations of key genes involved in the GA-mediated fruit development, several candidate genes were selected for RT-qPCR analysis, including *EVM0014035* (*PP2C51*), *EVM0018389* (*IAA12*), *EVM0039583*(*GH3.1*), *EVM0040143*(*ARR3*), *EVM0004038*(*SAPK3*), *EVM0042607*(*SAPK2*), *EVM0040808*(*GIDIB*), *EVM0014457*(*GIDIB*), *EVM0029677*(*GID2*). The results demonstrated that GA3 + 4 + 7 treatment significantly influenced the transcript levels of the examined genes associated with hormone-related pathways, in comparison to their corresponding controls. As anticipated, the expression trends of these candidate DEGs were consistent with the transcriptomic results (Fig. 7). The correlation coefficient between RNA-seq and RT-qPCR was found to be 0.7987 (Fig. S6), indicating the reliability of the experimental data obtained from transcriptomics.

## Discussion

Fruit size is a key factor influencing pear quality and consumer acceptance. In agricultural practices, the application of GA is prevalent for facilitating plant development. The application of gibberellin on grapes, apples, and rambutans has been shown to result in larger fruits, effectively enhancing their yield and quality [23–25]. In this study, our results indicated that treatments involving GA4 + 7 and GA3 + 4 + 7 significantly increased the fruit aspect diameter in accordance with previous findings. Notably, the combination of GA3 + 4 + 7 was found to be the most effective at improving pear fruit size by primarily promoting elongation of the longitudinal diameter. This observation partially diverges from previous studies and may be attributed to variations in cultivation conditions as well as environmental factors [26]. Additionally, research has shown that GA has a dual role in fruit development. It not only stimulates cell elongation in fruit tissues but also promotes cell division [27–30]. The research confirms that following treatment with GA3 + 4 + 7 during the early stages of fruit development, there is a predominance of flesh cell division which leads to an increase in total flesh cell number. Overall, this study demonstrates that the application of GA significantly enhances the enlargement of ‘Cuiguan’ pear fruit, indicating that GA plays a crucial role in the developmental process of pear fruit, with certain combinations of GA exhibiting particularly effective outcomes. Further research is necessary to refine and optimize the application of GA in pear cultivation to maximize both yield and consumer satisfaction.

Plant hormones play a crucial role in regulating plant development. It has been reported that the application of exogenous GA not only regulates plant development but also affects the synthesis of endogenous hormones such as auxin, cytokinin, and abscisic acid, along with their associated expression [13, 31]. In this study, GO enrichment analysis and KEGG enrichment analysis revealed significant enrichment in pathways associated with plant hormone synthesis and signal transduction. Exogenous application of GA resulted in alterations to endogenous GA levels by down-regulating *PyKAO1-like* (*EVM0011716*, *EVM0031581*), *PyKAO2-like* (*EVM0010061*), and *PyGA20ox1-like* (*EVM0034050*) within 24 h post treatment, as well as *PyGA3ox1-like* (*EVM0028197*) at 12 h post treatment. Similar downregulation of *GA20ox-like* and *GA3ox-like* has been reported in *Paphiopedilum orchids* following GA3 treatment at three developmental stages [32], as well as for *SlKAO2* and *SlGA20ox4* in tomato fruit after exogenous supplementation with GA4 treatment [33]. Notably, the gene *PyGA2ox1-like* (*EVM0004511*, *EVM0013841* and *EVM0010279*), which is involved in degrading active GA within the gibberellin metabolic pathway, exhibited upregulation during the initial 12-hour period post treatment. This observation aligns with findings demonstrating regulation of GA content through enhanced expression of the genes encoding gibberellin 2-oxidase 1 proteins (GA2ox1) following GA3 application in *Oenanthe javanica* (Blume) DC [34]. These observations are consistent with reports that exogenous applications of GA regulate fruit growth and development by modulating the expression of genes within the gibberellin signal transduction pathway [35, 36].

KEGG enrichment analysis of genes involved in plant hormone signal transduction pathways in response to exogenous GA3 + 4 + 7 treatment indicated alterations in the expression levels of genes related to auxin, cytokinin, ethylene and abscisic acid (Fig. 4). Previous studies have demonstrated that in carrots, tomato and grape, following the application of GAs, the transcriptional levels of genes related to gibberellin and other phytohormones such as auxin, cytokinin, and abscisic acid-vary depending on fluctuations in GA content [32–34, 37]. Auxin and gibberellin frequently collaborate to drive cell expansion and division, processes that are essential for fruit growth. These hormones can activate genes that are involved in cell wall loosening and remodeling, thereby facilitating cell expansion, especially during the early stages of fruit development when rapid cell growth is crucial. Both auxin and GA can influence the expression of key transcription factors and downstream target genes. Auxin-responsive genes, such as those encoding ARF/IAA proteins, can interact with GA signaling components to modulate the activity of transcription factors like DELLA proteins [8]. This cross-regulation ensures precise control of gene expression is involved in fruit development. In particular, in fruits such as tomato, apple, and citrus, auxin and gibberellin have been shown to interact with each other to drive fruit growth and development [38–41]. Furthermore, the transcriptional activity related to gibberellin as well as auxin, cytokinin, ethylene, and abscisic acid shows notable change upon administering exogenous GAs. Therefore, GA may interact with other hormonal pathways through crosstalk mechanisms that regulate pear fruit development. Our study concentrated on examining the expression levels of phytohormone associated genes within fruits; however, further investigation is required to elucidate these genes’ specific functions across various tissues within pears.

To elucidate the mechanisms underlying fruit size development, numerous genes associated with cell division, cell expansion, and cell cycle have been identified. These include cytochrome P450 genes, F-box proteins, kinesins, genes implicated in cell EXP, cell wall modifying enzyme XTH, CDKs, two cyclin genes, genes encoding TIP, and various membrane proteins. Cyclin-like genes and kinesin-like proteins that we have identified are primarily affect fruit size by modulating the progression of the cell cycle [42, 43]. Research indicates that kinesin genes *CsKF1* and *CsKF7* positively correlate with the rate of cell expansion and respond to plant hormones, suggesting their potential within plant hormone signaling pathways [44]. The wheat cytochrome P450 protein TaCYP78A3 positively regulates seed size by facilitating an increase in cell number [45]. The F-box protein SAP is a component of the SKP1/Cullin/F-box E3 ubiquitin ligase complex, which promotes cell division in meristematic tissue and consequently regulates organ size [46]. The LONGIFOLIA (LNG) related gene in *Arabidopsis* activates XTH, thereby controlling cell polar elongation [47]. Furthermore, tonoplast intrinsic protein enhances water absorption and vacuolar expansion during the germination of broad beans, leading to a rapid cell elongation [48]. Certain expansion proteins found in potato tubers and stem tissues can induce cell expansion, resulting in the extension of cell wall; notably, some of these genes are also regulated by hormones [49]. Membrane proteins are crucial for regulating intracellular signal transduction, with many forming clusters that initiate downstream signaling pathways to modulate various aspects of cell behavior, including migration, proliferation, and survival [44]. Our result revealed that genes associated with the cell cycle, cell expansion, and cell division exhibit significant expression variations following exogenous GA treatment, this includes several DEGs involved in hormonal regulation and cellular signaling transduction.

Transcription factors (TFs) play a pivotal role in plant growth and development. Our analysis of the DEGs dataset revealed that a total of 702 TFs exhibited differential expressions in the fruit flesh following exogenous GA3 + 4 + 7 treatment. Prior studies have demonstrated that certain members of specific gene families are involved in regulating plant organ size [50–54]. Integrating the findings from previous studies reveals that these TFs are likely involved in the development processes of pear fruits; however, their precise transcriptional regulatory mechanisms warrant further exploration. Furthermore, we employed WGCNA to investigate relationships among several plant hormone-related genes alongside genes implicated in DNA replication and TF-encoding genes. Ultimately, our findings suggest that these genes are collectively involved in the GA-regulated fruit development processes and there is a certain degree of crosstalk mechanism.

## Conclusions

This study investigated the effects of exogenous GA on pear fruit growth and development using morphological, cytological, and transcriptomic analyses. The results showed that treatment with GA3 + 4 + 7 significantly promoted fruit elongation, increased cell number, enhanced cell division, and increased fruit weight. Transcriptomic analysis revealed a complex regulatory network involving differentially expressed genes related to cell division, expansion, and hormone signaling pathways. GA not only affected genes involved in GA biosynthesis and signaling but also altered the expression of genes in other hormone pathways, highlighting the interplay among plant hormones. Our findings provide new insights into the molecular mechanisms underlying fruit development and establish a theoretical basis for improving fruit quality through genetic or chemical approaches.

## Supplementary Information

Supplementary Material 1: Fig. S1 Phenotypic characteristics of pear fruits treated with different exogenous GAs over 0–80 DAT.

Supplementary Material 2: Fig. S2 Statistics of differentially expressed genes (DEGs) upregulated and downregulated before and after GA3+4+7 treatment.

Supplementary Material 3: Fig. S3 GO enrichment analysis of DEGs between GA-treated and control groups at different time points.

Supplementary Material 4: Fig. S4 KEGG enrichment analysis of DEGs between GA-treated and control groups at different time points.

Supplementary Material 5: Fig. S5 Differentially Expressed Genes Related to the Auxin Signaling Pathway after GA Treatment.

Supplementary Material 6: Fig. S6 Consistency analysis between RNA-seq data and RT-qPCR results.

Supplementary Material 7: Table S1. Sequence of primers used for RT-qPCR in this study.

Supplementary Material 8: Table S2. Effect of GA treatment and Control on pear fruit.

Supplementary Material 9: Table S3. Summary of the sequence data analysis.

Supplementary Material 10: Table S4. Summary of RNA-Seq map.

Supplementary Material 11: Table S5. The transcript levels of GA-related genes in pear under GA treatment.

Supplementary Material 12: Table S6. The transcript levels of fruit size - related genes in pear under GA treatment.

## Data Availability

The RNA-seq analysis has been obtained from the NCBI repository under accession number PRJNA1230817. All data generated or analyzed during this study are included in this published article and its supplementary information files.

## Abbreviations

GA: Gibberellin
CK: Control
T: Treatment
RNA-seq: RNA Sequencing
DEGs: Differentially Expressed Genes
RT-qPCR: Real Time Quantitative-PCR
POS1: Physalis Organ Size 1
QTL: Quantitative Trait Locus
CLV-WUS: CLAVATA- WUSCHEL
CPS: Ent-Copalyl Diphosphate Synthase
KS: Ent-Kaurene Synthase
KO: Ent-Kaurene Oxidase
KAO: Ent-Kaureneoic Acid Oxidases
GID1: GA Insensitive Dwarf 1
SCF: SKP1-Cullin 1-F-box Protein
DAFB: Days After Full Blooming
DAT: Days After Treatment
PCA: Principal Component Analysis
FPKM: Fragment Per Kilobase of Transcript Per Million Mapped Reads
FDR: False Discovery Rate
GO: Gene Ontology
KEGG: Kyoto Encyclopedia of Genes and Genomes
Fw: Fruit Weight
AUX1: Auxin-Influx Carrier
TIR1: Transport Inhibitor Response 1
AUX/IAA: AUXIN/Indole-3-Acetic Acid
ARF: Auxin Response Factor
GH3: Gretchen Hagen 3
SAUR: Small Auxin-up RNA
EIN3: Ethylene Insensitive 3
EBF1: EIN3-Binding F-box Protein 1
ERF1: Ethylene-Responsive Transcription Factor 1
AHP: Histidine-Containing Phosphotransfer
ARR: Arabidopsis Response Regulator
PYR/PYL: Pyrabactin Resistance 1-like
PP2C: Phosphatase 2C
SnRK: Sucrose Non-Fermenting Related Protein Kinase
ABF: ABA Responsive Element Binding Factor
EXP: Expansion
XTH: Xyloglucan Endotransglucosylase/Hydrolase
CDKs: Cyclin-Dependent Kinases
TIPs: Tonoplast Intrinsic Proteins
WGCNA: Weighted Gene Co-Expression Network Analysis
LNG: LONGIFOLIA
TFs: Transcription Factors
bHLH: Basic Helix-Loop-Helix
MYB: MYB Transcription Facto
NAC: NAM, ATAF, and CUC
NAM: No Apical Meristem
ATAF: Arabidopsis Transcription Activation Factor
CUC: Cup-shaped Cotyledon
WRKY: WRKY Transcription Factor
bZIP: Basic Leucine Zipper
C2H2: Cysteine2/Histidine2
HD-ZIP: Homeodomain Leucine Zipper
GRAS: GRAS Transcription Factor
Dof: DNA Binding with One Finger
