# Supplementary figures and images for: Transcriptome analysis reveals the role of GA in fruit development of ‘Cuiguan’ pear (Pyrus pyrifolia)
Source: BMC Plant Biol. 2025 Dec 9;26:71. doi: 10.1186/s12870-025-07854-3 (PMC12801501; doi:10.1186/s12870-025-07854-3)

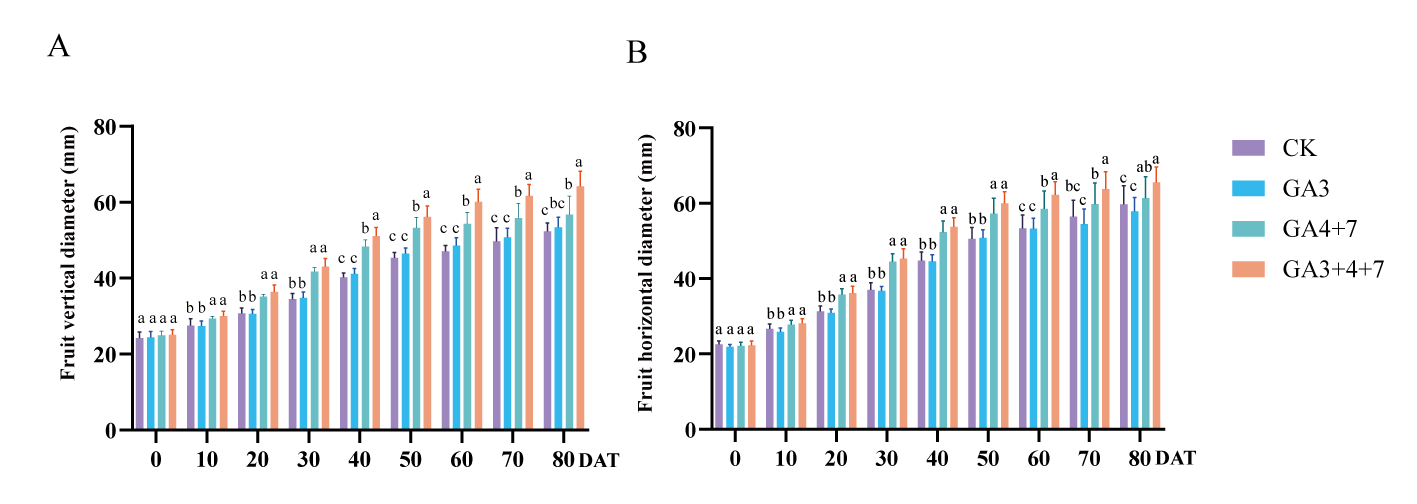

Supplement: Supplementary file 1 — Supplementary Material 1: Fig. S1 Phenotypic characteristics of pear fruits treated with different exogenous GAs over 0–80 DAT. [file 12870_2025_7854_MOESM1_ESM.tif]

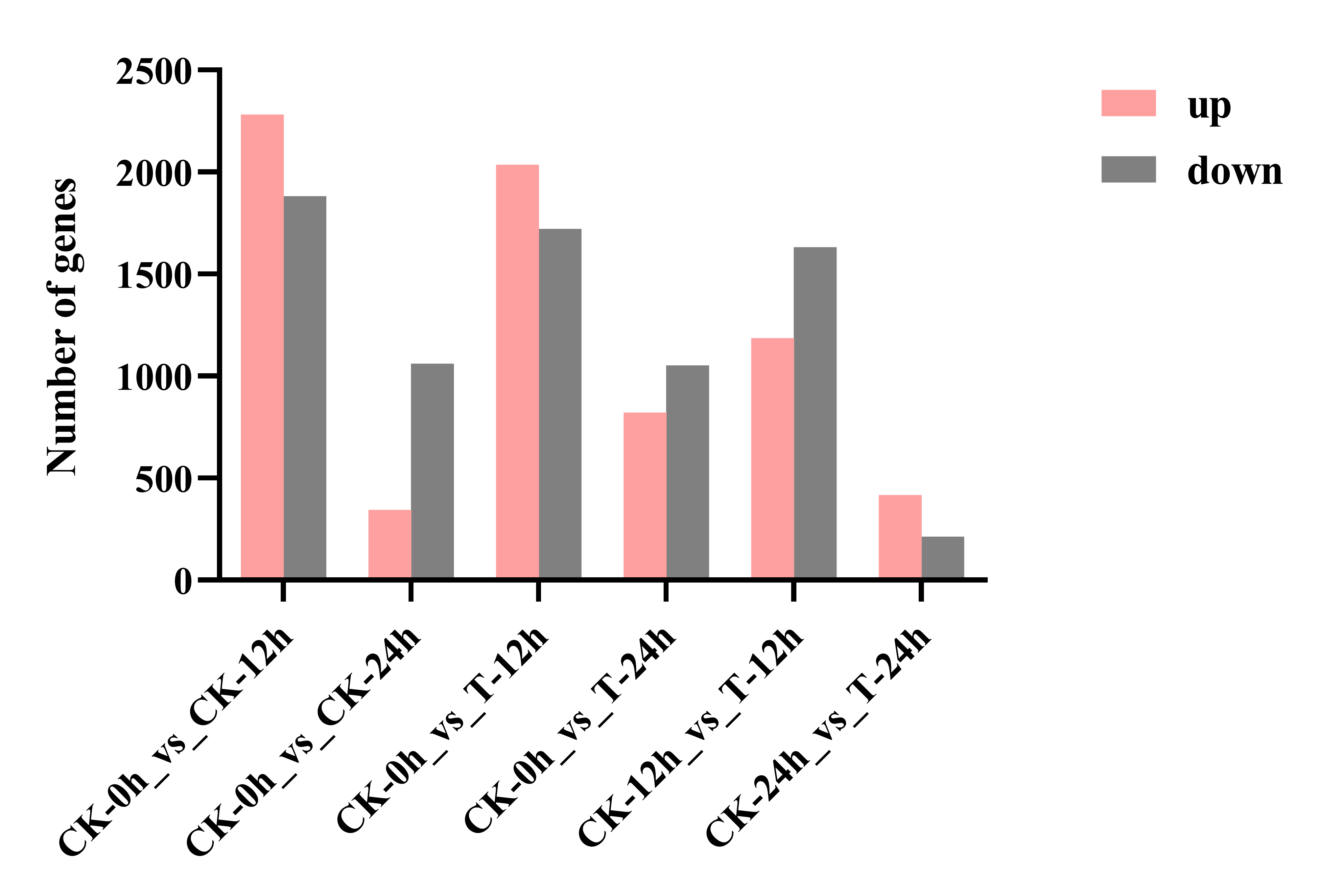

Supplement: Supplementary file 2 — Supplementary Material 2: Fig. S2 Statistics of differentially expressed genes (DEGs) upregulated and downregulated before and after GA3+4+7 treatment. [file 12870_2025_7854_MOESM2_ESM.tif]

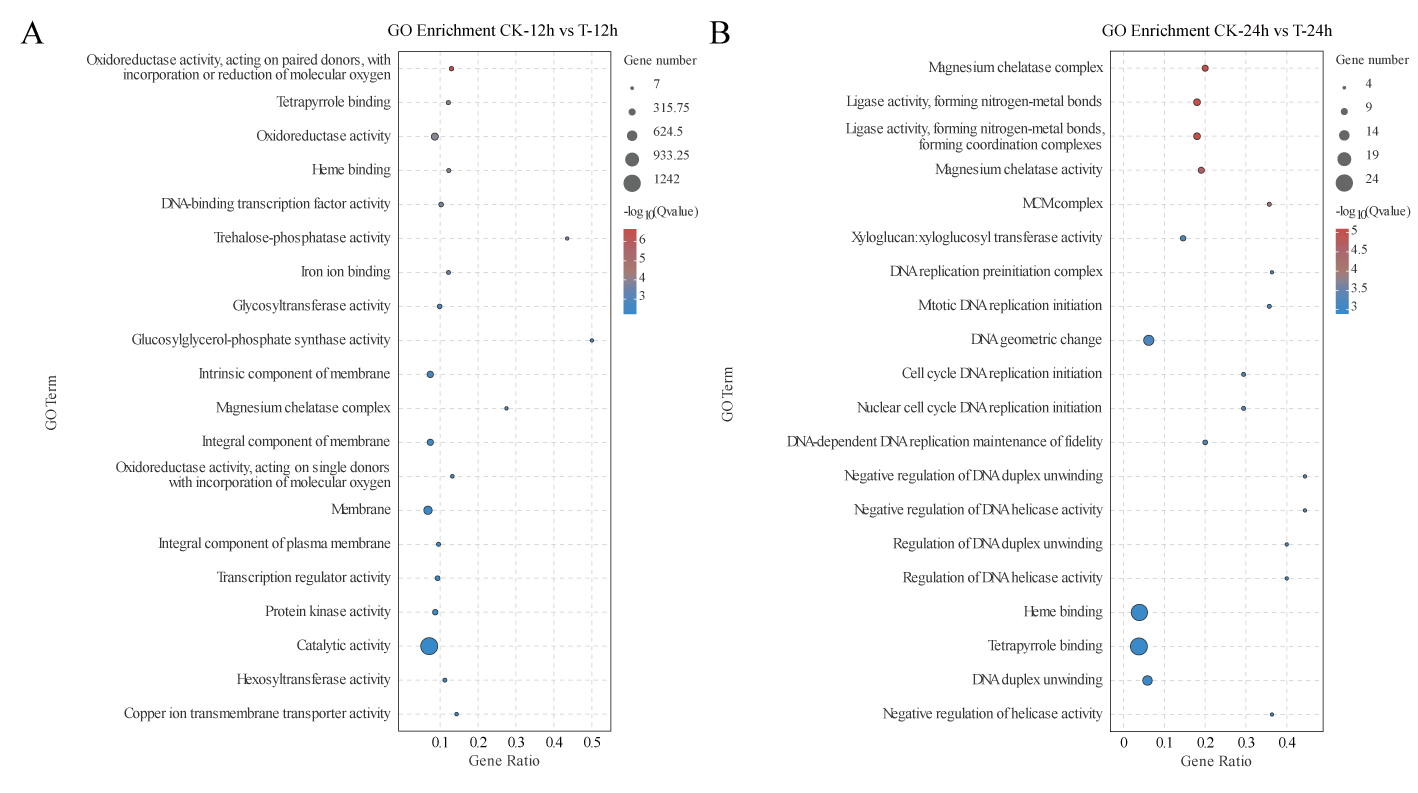

Supplement: Supplementary file 3 — Supplementary Material 3: Fig. S3 GO enrichment analysis of DEGs between GA-treated and control groups at different time points. [file 12870_2025_7854_MOESM3_ESM.tif]

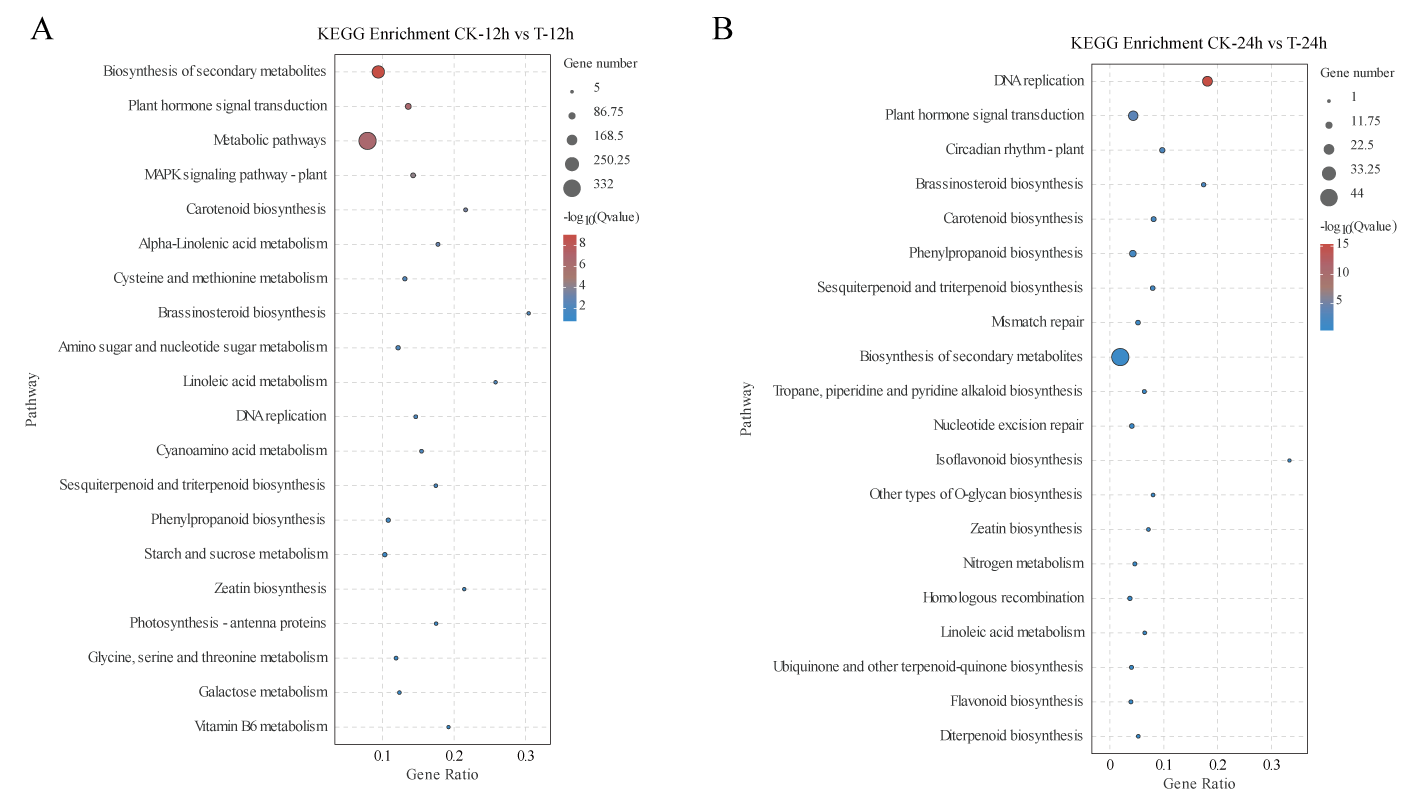

Supplement: Supplementary file 4 — Supplementary Material 4: Fig. S4 KEGG enrichment analysis of DEGs between GA-treated and control groups at different time points. [file 12870_2025_7854_MOESM4_ESM.tif]

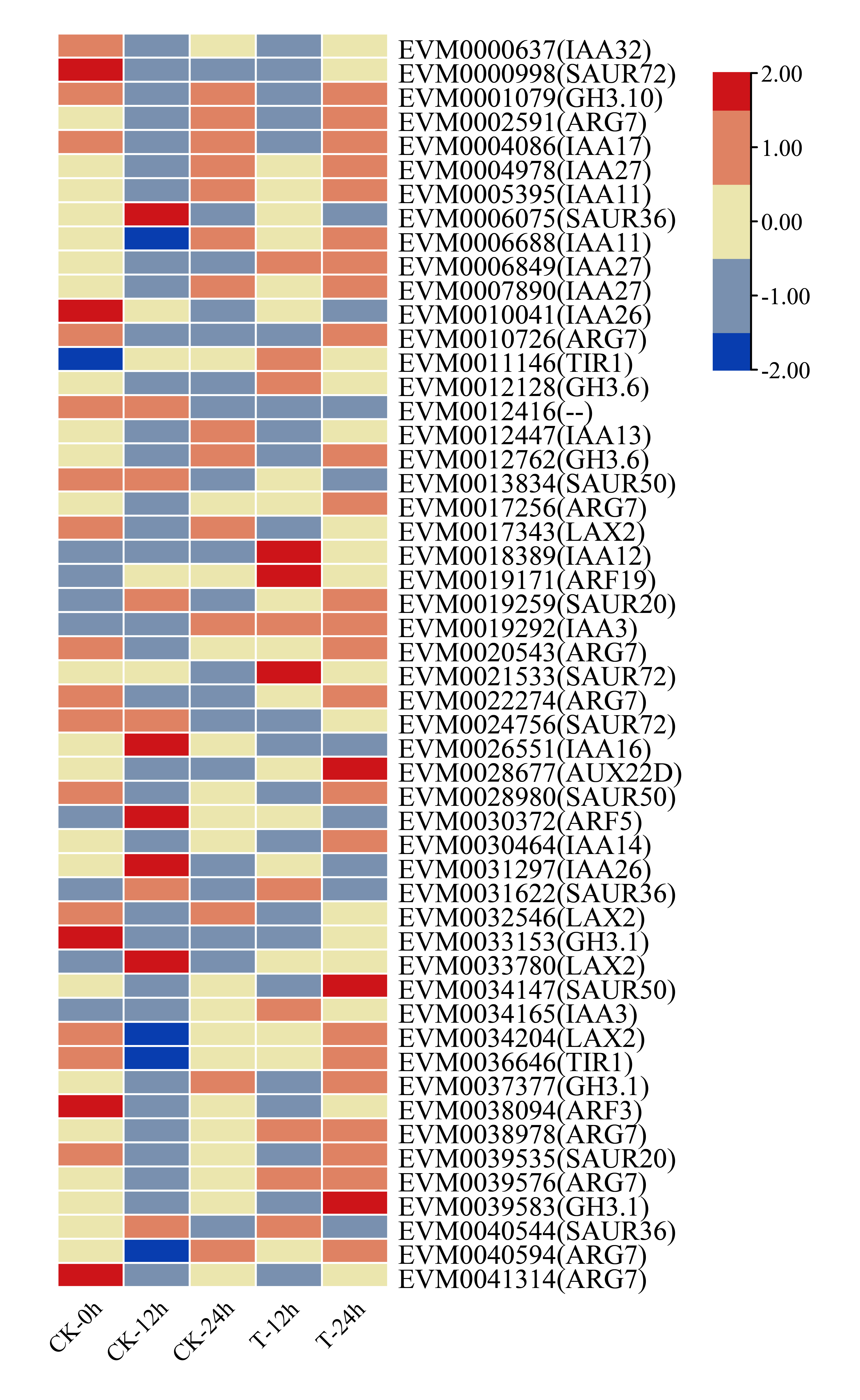

Supplement: Supplementary file 5 — Supplementary Material 5: Fig. S5 Differentially Expressed Genes Related to the Auxin Signaling Pathway after GA Treatment. [file 12870_2025_7854_MOESM5_ESM.tif]

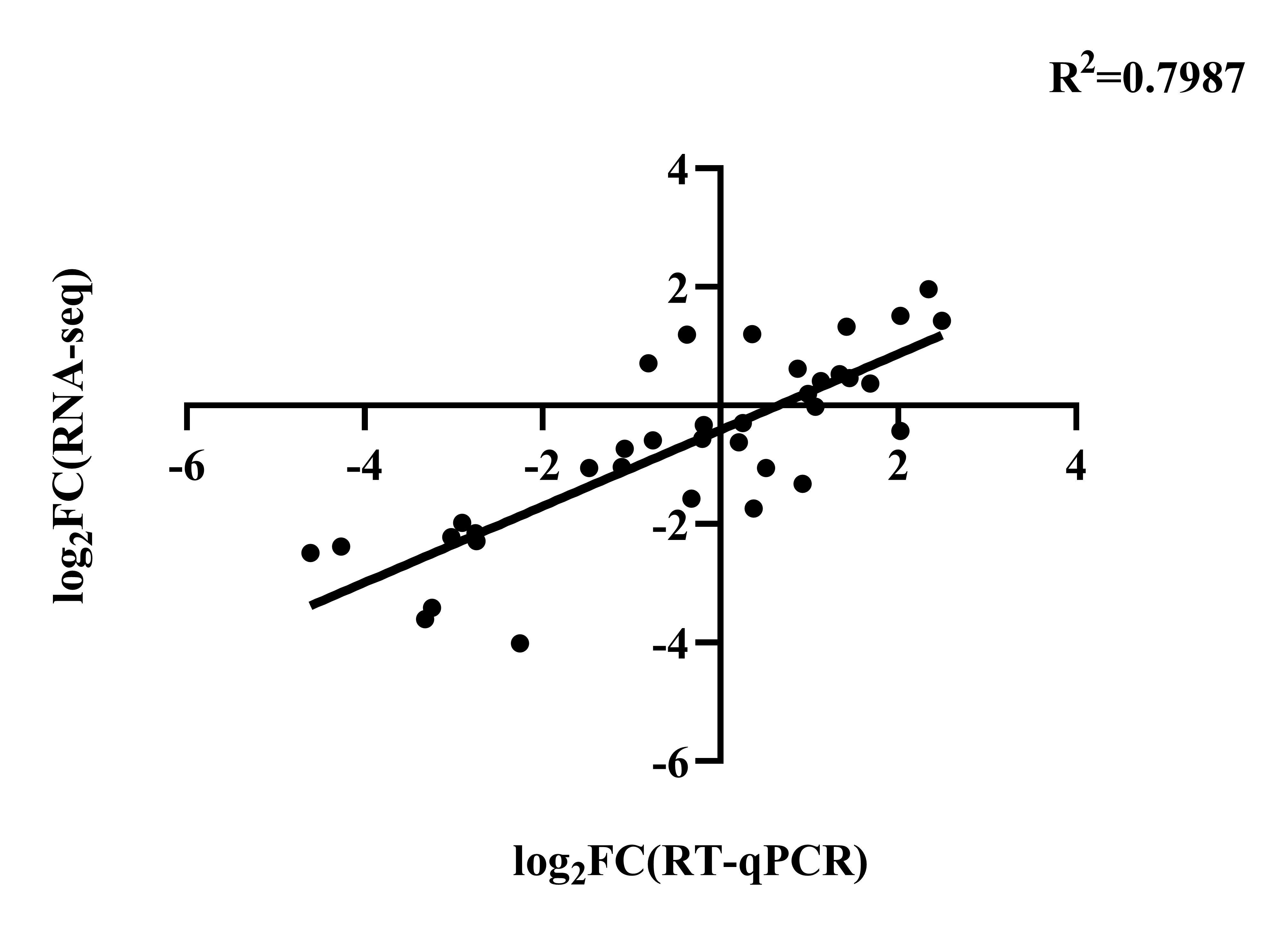

Supplement: Supplementary file 6 — Supplementary Material 6: Fig. S6 Consistency analysis between RNA-seq data and RT-qPCR results. [file 12870_2025_7854_MOESM6_ESM.tif]
